# Proliferative glomerulonephritis with monoclonal immunoglobulin deposits and atypical pathological findings treated with corticosteroid and rituximab

**DOI:** 10.1007/s13730-023-00813-7

**Published:** 2023-08-07

**Authors:** Machi Mori, Akihito Tanaka, Kayaho Maeda, Shoji Saito, Kazuhiro Furuhashi, Shoichi Maruyama

**Affiliations:** 1 Department of Nephrology, Nagoya University Hospital, Nagoya, Aichi Japan; 2 Department of Nephrology, Nagoya University Graduate School of Medicine, Nagoya, Aichi Japan

**Keywords:** PGNMID, Proteinuria, Rituximab, Vaccination, Proliferative glomerulonephritis with monoclonal immunoglobulin deposits

## Abstract

A 16-year-old girl with fever that appeared after taking the second COVID-19 vaccine presented to the clinic with a serum creatinine of 0.89 mg/dL and C-reactive protein of 6.9 mg/dL. She had proteinuria and microscopic hematuria, with slowly worsening kidney function. Her kidney biopsy showed fibrocellular crescents in seven of nine glomeruli that were observed under light microscopy. Another glomerulus showed endocapillary hypercellularity and mesangial cell proliferation. Electron-dense deposits were significant in the mesangial area, with monoclonal IgG1-κ and C3 deposition by immunofluorescence. The patient was diagnosed with proliferative glomerulonephritis with monoclonal immunoglobulin deposits (PGNMID) and atypical pathological finding of diffuse crescent formation. The treatment regimen for PGNMID has not yet been established, and the appropriate duration of treatment is unknown. In our case, considering that rituximab acts by binding to CD20 on the surface of B cells through its crystallizable fragment, it was administered in addition to prednisolone, which successfully decreased the proteinuria over time.

## Introduction

Proliferative glomerulonephritis with monoclonal immunoglobulin deposits (PGNMID) is a rare form of glomerulonephritis (GN) characterized by the presence of glomerular monoclonal immunoglobulin (Ig) deposits [1]. Patients younger than 20 years of age with PGNMID usually present with variable degrees of proteinuria, hematuria, and kidney insufficiency. The underlying monoclonal Igs are often undetectable by serum or urine protein electrophoresis. Membranoproliferative GN pattern with monoclonal IgG deposits and immune complex-type electron-dense deposits (EDDs) are common. However, the pathogenesis of PGNMID remains unclear. It has been suggested that circulating monoclonal IgG molecules may lead to glomerular injuries once deposited in glomeruli and, in turn, activate complement cascade causing inflammatory reactions [2]. Several case reports have shown that the COVID-19 vaccine may cause GN. Thus, it is necessary to consider that this case of PGNMID represents the possibility of kidney injury induced by the COVID-19 vaccine. In this case, we encountered PGNMID characterized by monoclonal IgG1-κ deposition with crescent formation in the majority of the glomeruli after receiving the COVID-19 vaccine.

## Case report

In November 2021, a 16-year-old girl who received the second COVID-19 vaccine 2 months prior to her visit to another hospital presented to her previous physician with fever. She had no medical history, and no abnormal urinalysis findings were noted. No other prodromal symptoms such as infection were observed. Regarding her family history, her aunt has systemic lupus erythematosus (SLE). Blood tests did not reveal a specific cause of the fever, except for elevated creatinine (0.89 mg/dL) and C-reactive protein (6.9 mg/dL). Although the fever subsided, the patient had recurrent fever, as well as gross hematuria and proteinuria, which worsened over time. On December 9th, she developed macroscopic hematuria and was referred to our hospital with suspected nephritis.

### Physical examination on admission

164.0 cm, 52.1 kg, 36.8 °C, 122/61 mmHg, 70 bpm, SpO_2_ 98% (room air).

Glasgow Coma Scale E4V5M6.

Clear respiratory and heart sounds.

No abdominal tenderness.

No skin rash.

No joint pain.

No peripheral edema.

### Laboratory data on admission:

Table 1 shows the results of blood examination performed on admission.

**Table 1:** Laboratory examination on admission

WBC (/μL)	5800
Hb (g/dL)	11.7
PLT (10^4^/μL)	21.2
TP (g/dL)	7.0
Alb (g/dL)	3.9
AST (U/L)	17
ALT (U/L)	8
ALP (U/L)	73
BUN (mg/dL)	22.9
Cre (mg/dL)	0.97
Na (mEq/L)	142
K (mEq/L)	4.0
CRP (mg/dL)	0.05
Anti-nuclear antibody	< 40
Anti-DNA Ab (IU/mL)	4.4
Anti-Sm Ab (U/mL)	< 10
MPO-ANCA (IU/mL)	< 3.5
PR3-ANCA (IU/mL)	< 3.5
Anti-GBM Ab (U/mL)	< 2.2
Treponema pallidum Ab	Negative
Serologic test for syphilis RPR	Positive
CH50 (U/mL)	54.5
C3 (mg/dL)	104.5
C4 (mg/dL)	27.4
ESR (mm)	19
PT (%)	104.3
INR	0.98
APTT (%)	94.6

*WBC* white blood cell count, *Hb* hemoglobin, *PLT* platelet count, *TP* total protein, *Alb* albumin, *AST* aspartate aminotransferase, *ALT* alanine aminotransferase, *ALP* alkaline phosphatase, *BUN* blood urea nitrogen, *Cre* creatinine, *Na* sodium, *K* potassium, *CRP* C-reactive protein, *DNA* deoxyribonucleic acid, *Ab* antibody, *MPO* myeloperoxidase, *ANCA* anti-neutrophil cytoplasmic antibody, *PR3* proteinase-3, *GBM* glomerular basement membrane, *RPR* rapid plasma reagin latex agglutination test, *CH50* complement total, *C3* complement 3, *C4* complement 4, *ESR* erythrocyte sedimentation rate, *PT* prothrombin time, *INR* international normalized ratio, *APTT* activated partial thromboplastin clotting time

Table 2 shows the urinalysis results.

**Table 2:** Clinical course of urinalysis

	#2021.12.16	#2021.3.17	#2022.4.26	#2022.12.17	#2023.5.17
Proteinuria	(1+)	(2+)	(2+)	(2+)	(3+)
Hematuria	(1+)	(3+)	(3+)	(1+)	(1+)
Protein/Cr ratio (g/gCr)	0.3	1.50	1.40	0.79	0.72
RBC	5–9/HPF	30–49/HPF	5–9/HPF	1–4/HPF	1/HPF <
Dysmorphic RBC	Negative	A few	Negative	Negative	Negative
β2MG (μg/L)	159			341	31
NAG (U/L)	4.5			6.5	13.8

*Cr* creatinine, *RBC* red blood cell, *β2MG* beta-2-microglobulin, *NAG* *N*-acetyl-beta-d-glucosaminidase

**Fig. 1:**
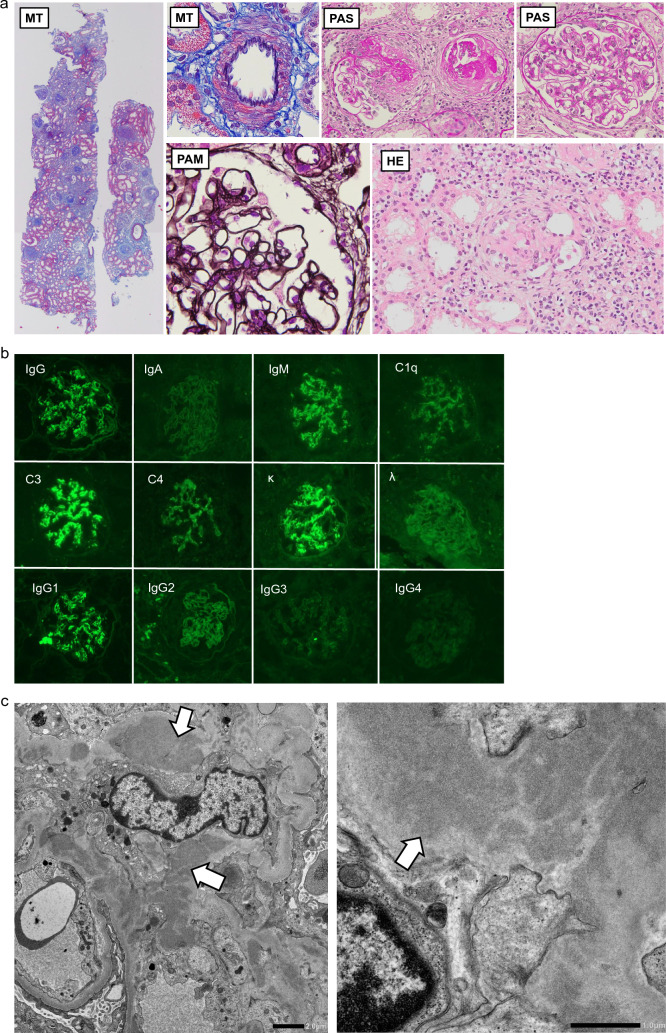
**a** Findings from light-microscopy. The left row panel shows low-power view of MT with original magnification ×40. The middle upper panel of MT shows the arteriole with original magnification ×400. The middle and right row upper panels of PAS show glomeruli with original magnification ×100. The middle row lower panel of PAM shows the glomerular basement membrane with original magnification ×400. The right row lower panel of HE shows the cells infiltrating the interstitium of kidney. *MT* Masson trichrome stain, *PAS* periodic acid–Schiff stain, *PAM* periodic–acid methenamine silver stain, *HE* hematoxylin–eosin stain. **b** Findings from immunofluorescence microscopy. **c** Findings from electron microscopy. The arrows show electron dense deposits. The left panel is a weakly magnified image and the right panel is a strongly magnified image

The patient’s history of fever that lasted 3–4 days every month and her family history suggested SLE as one of the differential diagnoses; however, physical examination and blood test results did not meet the diagnostic criteria for SLE. In March 2022, we performed a kidney biopsy; the results are shown in Fig. 1a–c.

### Findings of kidney biopsy

Light microscopy: two pieces were obtained from the cortex; 19 glomeruli were observed, 10 of which were completely obstructed, seven of remaining glomeruli showed fibrocellular crescents, and one showed segmental sclerosis. Another glomerulus, without crescent formation, showed endocapillary hypercellularity and mesangial cell proliferation. No spike formation or bubbly appearance were observed. Arterial intimal thickening or inflammation was not observed. About 50% of the cortex showed interstitial fibrosis and tubular atrophy. A moderate inflammatory cellular infiltrate is seen around the globally sclerotic glomeruli, but it is predominantly mononuclear cells, with few neutrophils, eosinophils, or plasma cells. There is little significant tubulitis in the non-atrophic tubules, suggesting that the interstitial damage in this case is mainly secondary to glomerular injury.

Immunofluorescence (IF) microscopy: positivity for IgG, IgM, C3, and κ in the mesangial area and negativity for IgA, C1q, C4, and λ. Regarding the IgG subclass, positive findings were observed for only IgG1.

Electron microscopy: EDDs significantly seen in the mesangial area and partially in the subendothelium and basement membrane.

**Fig. 2:**
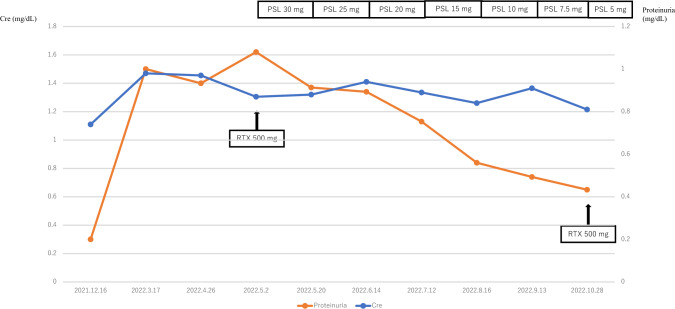
Clinical course of serum creatinine and proteinuria. *Cre* creatinine, *PSL* prednisolone, *RTX* rituximab

A kidney biopsy revealed a large amount of crescent formation, which was considered atypical for PGNMID; however, there seemed to be no other appropriate diagnosis, and we finally diagnosed PGNMID. The clinical course of the patient is shown in Fig. 2. The patient was treated with corticosteroids and rituximab, which led to decrease in urinary protein levels. Furthermore, macroscopic hematuria was no longer observed.

## Discussion

We encountered a case of PGNMID with a relatively high proportion of crescents after the COVID-19 vaccination.

This patient initially presented with repeated fever after the administration of the COVID-19 vaccination. She was also suspected to be serologically positive for syphilis. However, the patient was negative for treponema pallidum antibody, had no history of suspected syphilis, and was considered a biological false positive. Her blood tests were negative for anti-nuclear antibodies, no anti-DNA antibodies, and no decreased complement activity, and kidney biopsy results did not reveal any characteristics of SLE despite the presence of biological false positive for syphilis.

In the largest cohort of adult patients, PGNMID often displays a diffuse membranoproliferative or endocapillary proliferative GN pattern. Although approximately 30% of the cases show crescents, most are focal. IF microscopy shows glomerular deposits composed of a single light-chain isotype (75% *κ* and 25% *λ*) and a single heavy-chain subclass including IgG1 (28%), IgG2 (6%), and IgG3 (66%), respectively. Monoclonal IgG4 deposits are usually not detected. Electron microscopy generally reveals granular, EDDs with subendothelial and mesangial distributions. The IF pattern of the kidney biopsy in our patient was not frequently observed, nor was it extremely rare. Crescents are usually observed in approximately 30% of PGNMID cases [3, 4]. In our case, the proportion of crescents was very high, which did not exclude PGNMID but was thought to be atypical. Further, the fact that IgM was positive in IF also complicated the diagnosis. There are several case reports that SARS-CoV-2 vaccination is associated with the onset or relapse of nephritis. A review article has shown 27 clinical cases related to COVID-19 vaccines and GN; 13 cases of newly diagnosed GNs and 14 cases of relapse. IgA nephritis was the most common pathology followed by minimal change disease. Others included anti-glomerular basement membrane nephritis, ANCA vasculitis, and anti-phospholipase A2 receptor associated membranous nephropathy [5]. Another paper presents pathological findings of vaccine-associated nephritis [6]. In this paper, several cases of crescentic GN with IgM deposition in IF microscopy were reported. Taking this into account, our case could have been a combination of PGNMID and vaccine-induced crescentic GN. The mRNA vaccine against COVID-19 contains purified modified mRNA and a vehicle that delivers the mRNA into host cells. Once the vaccine is injected, mRNA is translated into a target protein, which, in turn, results in immune system activation, inducing neutralizing antibodies to a level far beyond that of convalescent serum [7, 8]. In this process, the neutralizing antibody cross-reacts with the SARS-CoV-2 spike protein, but other autoantibodies (transglutaminase 3, nuclear antigen, myelin protein, mitochondria, myosin, thyroid peroxidase, collagen, and claudin) are also triggered, causing a cross-reaction with the SARS-CoV-2 spike protein [9]. Therefore, it is highly likely that this high immunogenicity and cross-reactivity could lead to unexpected and perhaps nonspecific immune activation that may trigger autoimmune processes or unmask the underlying autoimmune diseases [10]. There are also reports that the characteristics of COVID-19 induced nephritis are atypical compared to those of commonly seen nephritis [11]. In our case, we considered that it was atypical as PGNMID, and the patient either had already developed PGNMID before the COVID-19 vaccine or it was triggered by the vaccination. However, even among us, the IgM deposition caused controversy in the diagnosis of PGNMID. We discussed and determined that atypical PGNMID with IgM deposition and many crescent formations developed with the addition of an atypical factor due to vaccination, as reported in [6], although it is unclear whether vaccination drove or triggered the onset of the disease. And we thought that there was no other mechanism that could clearly explain the present condition.

PGNMID is not frequently observed, and there is no established treatment. Because of the pathogenesis by which monoclonal antibodies are produced, treatments targeting B cells or plasma cells are often chosen. If multiple myeloma is diagnosed, treatment may be administered accordingly. In our case, no M protein was detected in the blood or urine and was only found in the kidney. In addition, no clinical symptoms or findings suggestive of multiple myeloma were observed.

Although there is no established treatment, medications that remove B cells (e.g., rituximab) have often been selected for PGNMID. It is difficult to infer whether a patient with non-IgM (e.g., IgG or IgA) monoclonal protein deposits in the kidney has a hypothetical plasma cell clone or B cell clone. In such patients, it is reasonable to initiate empirical treatment with either plasma cell- or B cell-directed therapies. Treatment should be individualized based on disease severity and patient preferences. For example, some experts may take a stepwise approach and start by administering B-cell-directed therapy, which may be associated with fewer adverse effects, and then switch to plasma cell-directed therapy for patients who fail to respond to the initial therapy [12]. There have been several previous cases of PGNMID that were successfully treated with Rituximab [13]. Rituximab is a human monoclonal antibody with specific affinity for CD20, a B-lymphocyte transmembrane protein expressed on peripheral and malignant B cells. It has been approved for the treatment of blood B-cell malignancies and non-hematologic B-cell-mediated diseases, particularly autoimmune disorders. It also has the ability to activate different effector functions such as direct antitumor effects via apoptosis or other cell death pathways, antibody-dependent cell phagocytosis, complement-dependent cytotoxicity, and antibody-dependent cell-mediated cytotoxicity. The mechanism of action involves the binding of rituximab to CD20 on the surface of B cells through its crystallizable fragment (Fc). Following this interaction, CD20 is reorganized into lipid rafts, which are subdomains of the plasma membrane containing high concentrations of cholesterol and glycosphingolipids. Lipid raft formation triggers the complement cascade and increases phagocytosis. Antibody-dependent cell-mediated cytotoxicity occurs after the interaction of the Fc portion of rituximab with Fcγ receptors expressed on the surface of effector cells (macrophages, granulocytes and natural killer cells), causing the release of cytokines, chemokines and mediators that can kill target cells [14].

In our case, we chose a combination of prednisolone and rituximab therapy considering the aforementioned mechanism of action. Steroids have rapid therapeutic anti-inflammatory effects and were first administered when the patient exhibited severe crescent formation. To suppress monoclonal antibody production, we administered rituximab after discussion and approval by the Institutional Review Board, as rituximab is not covered by medical insurance against PGNMID in Japan. As a result, the proteinuria decreased, and we believe that the course of treatment was relatively effective. However, there is no further established information regarding the duration of treatment; thus, additional investigations are necessary. In our patient, acute-phase lesions were observed along with renal tubular atrophy and interstitial deterioration, which are typically observed in chronic cases. Thus, we believe that complete recovery is difficult to achieve, and the duration of treatment needs to be discussed. In the present case, clinical findings of the acute phase were observed, and treatment was aimed to control these findings. Initially, the second dose of rituximab was administered at an interval of 6 months so that there would be no time when the effect of rituximab would wear off. However, the timing of the administration of the drug in the future needs to be discussed.

In conclusion, we encountered an atypical PGNMID case with a relatively high proportion of crescent formation that developed after COVID-19 vaccination. Although there is no established treatment, rituximab was effective in this case. The treatment duration should be discussed.
